# Long-term survival in patients with PMP: a single-institutional retrospective study from China

**DOI:** 10.1186/s12957-023-03232-1

**Published:** 2023-10-28

**Authors:** Rui Yang, Yu-Bin Fu, Xin-Bao Li, Ru Ma, Yan-Dong Su, He-Liang Wu, Xin-Li Liang, Yan Li

**Affiliations:** 1 Department of Peritoneal Cancer Surgery, Beijing Shijitan Hospital, Capital Medical University, Beijing, 100038 China; 2 Department of Peritoneal Cancer Surgery, Beijing Shijitan Hospital, Peking University Ninth School of Clinical Medicine, Beijing, 100038 China; 3 Department of Surgical Oncology, Beijing Tsinghua Changgung Hospital, Tsinghua University, Beijing, 102218 China

**Keywords:** Pseudomyxoma peritonei, Cytoreductive surgery, Hyperthermic intraperitoneal chemotherapy, Long-term survival, Completeness of cytoreduction, Pathological diagnosis

## Abstract

**Background:**

As the standard treatment for pseudomyxoma peritonei (PMP), cytoreductive surgery (CRS) plus hyperthermic intraperitoneal chemotherapy (HIPEC) can significantly prolong the survival of PMP patients, and some patients can even achieve long-term survival (LTS) or clinical cure. The purpose of this study was to analyze the clinicopathological and treatment features of PMP patients with LTS and to explore the survival benefit factors of PMP patients.

**Methods:**

The clinicopathological and prognostic data of PMP patients who received CRS + HIPEC at our center from December 2004 to May 2023 were retrospectively analyzed. PMP patients were divided into LTS group (≥ 10 years) and short-term survival (STS) group (< 5 years) according to the length of natural history. Univariate and multivariate analyses were performed to explore the beneficial factors of PMP patients with LTS.

**Results:**

A total of 609 patients with PMP received CRS + HIPEC treatment at our center. Two-hundred one patients with PMP were included in the study after screening, including 39 patients (19.4%) in the LTS group and 162 patients (80.6%) in the STS group. In STS group and LTS group, median overall survival based on natural history was 29.2 (2.4–59.9) *vs.* 138.9 (120.3–416.7) months. Univariate analysis revealed 8 factors (*P* < 0.05) with statistically significant differences between the two groups: gender, chemotherapy history, previous surgical score, Karnofsky Performance Status score, pathological diagnosis, lymphatic metastasis, peritoneal cancer index, and completeness of cytoreduction (CC). Multivariate analysis identified only two factors independently associated with LTS of PMP patients: CC and pathological diagnosis.

**Conclusion:**

Complete CRS and pathological features are two key factors affecting LTS in PMP patients.

## Introduction

Pseudomyxoma peritonei (PMP) is a malignant clinical syndrome characterized by the accumulation and redistribution of mucus produced by mucinous tumor cells in the abdominal cavity [1]. The concept of PMP was first proposed by Werth in 1884 to describe a patient with gelatinous mass in the abdominal cavity due to rupture of ovarian mucinous adenoma [2]. Most PMP originates from mucinous tumors of the appendix and a small part from primary mucinous tumors of ovaries, colon, and other organs [3]. According to epidemiological studies related to PMP, the incidence of PMP is about 2–4 cases in 1 million per year, and the prevalence is approximately 25.1 cases in 1 million, which is a rare disease [1, 4–6].

The clinical diagnosis of PMP is often accidental, such as in the imaging examination or surgical exploration for other surgical indications; the most common is acute appendicitis, inguinal hernia surgery, etc. Mucus builds up in the abdominal cavity with few symptoms in the early stages, until significant bloating leads to abdominal discomfort or difficulty breathing [7]. The compression of the internal organs and the inflammatory and fibrotic reaction of the mesothelium over time lead to intestinal obstruction, which is a fatal complication of untreated or recurrent PMP [8].

At present, the integrated treatment with CRS + HIPEC as the core has been the standard treatment for PMP. In 2020, PSOGI formally formulated international guidelines for the treatment of PMP with CRS + HIPEC [9]. According to literature reports, standardized CRS + HIPEC can significantly improve the overall survival of PMP patients up to 103.4–196 months, and the 5-year and 10-year overall survival rates can reach 49.0–92.1% and 32.8–80.8%, respectively [10].

The main purpose of this study was to analyze and compare the clinicopathological and treatment features between long-term survival (LTS) PMP patients and short-term survival (STS) PMP patients in our center.

## Patients and methods

### Patients

**Fig. 1:**
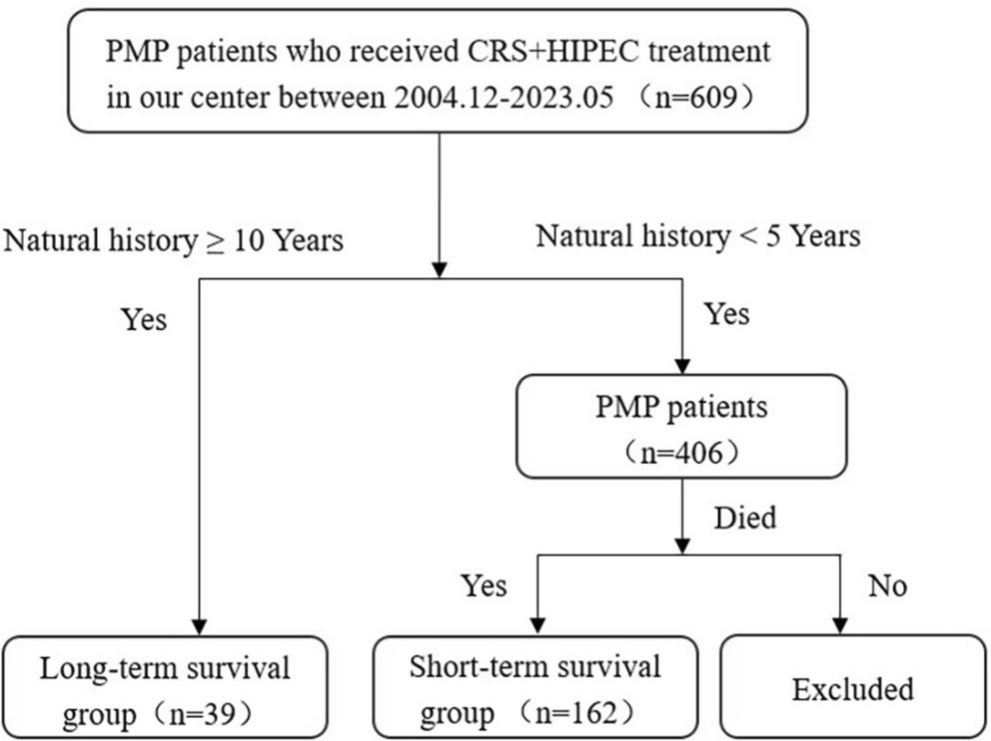
Selection of PMP patients

There were 609 PMP patients treated with CRS + HIPEC at Beijing Shijitan Hospital from December 2004 to May 2023. Data include pathological grade, peritoneal cancer index (PCI), completeness of cytoreduction (CC), operation duration, and serious adverse events (SAEs) 30 days after surgery, etc. PMP patients were divided into LTS group and STS group according to their length of natural history. The inclusion criteria for LTS group (Fig. 1) were as follows: (1) PMP was confirmed by pathology, (2) meeting the criteria of CRS + HIPEC [11], and (3) natural history ≥ 10 years. The inclusion criteria for STS group (Fig. 1) were as follows: (1) PMP was confirmed by pathology, (2) meeting the criteria of CRS + HIPEC [11], (3) natural history < 5 years, and (4) dead. This study was approved by the Institutional Review Board of Beijing Shijitan Hospital, Capital Medical University. All patients signed the informed consent.

### Natural history

The natural history in this study was defined as the period of time between the onset of disease-related symptoms and the deadline of follow-up or death in PMP patients.

### CRS + HIPEC

All CRS + HIPEC procedures were performed by the peritoneal metastasis specialist team of our center. After successful general anesthesia, a midline incision was made in the upper abdomen from the xiphoid process to the pubic symphysis to expose the abdominal cavity fully. And then, PCI score was comprehensively evaluated. After CRS, the CC score was evaluated based on the residual tumor size. Open HIPEC was administered after completion of CRS, with 120-mg cisplatin + docetaxel 120 mg or 120-mg cisplatin + mitomycin 30 mg at 43 °C for 60 min. Subsequently, functional reconstruction of digestive tract and abdominal closure were performed.

### Adverse events

Adverse events were defined as complications occurring within 30 days of CRS + HIPEC. According to Sugarbaker’s textbook on peritoneal metastasis [12], AEs are graded into five levels of severity: grade I, asymptomatic and self-limited; grade II, symptomatic and requiring medical treatment; grade III, requiring invasive intervention; grade IV, requiring ICU admission or reoperation; and grade V, postoperative death. Serious adverse events (SAEs) included grade III–V AEs.

### Statistical analysis

BM SPSS Statistics for Windows, version 26.0 (IBM Corp., Armonk, NY, USA) was used for data analysis. Measurement data are presented as median (range) or mean ± SD and analyzed by *t*-test or rank-sum test. Enumeration data were presented as frequencies and analyzed using the *χ*^2^ and Fisher’s exact tests. Univariate and logistic regression analyses were used to analyze the independent factors influencing the LTS of PMP patients.

## Result

### Major clinicopathological characteristics

A total of 201 PMP patients were included in this study, including 39 cases (19.4%) in the LTS group with natural history ≥ 10 years and 162 cases (80.6%) in the STS group with natural history < 5 years. In LTS and STS groups, female patients were 68 (42.0%) *vs.* 26 (66.7%) (*P* = 0.006), patients with chemotherapy history were 84 (51.9%) *vs.* 13 (33.3%) (*P* = 0.038), and patients with previous surgical scores of 2–3 were 107 (66.0%) *vs.* 18 (46.2%) (*P* = 0.016). The comparison of other major clinicopathological features between the two groups was shown in Table 1.

**Table 1:** Major clinicopathological characteristics of PMP patients between STS and LTS groups

Variable	STS group (*n* = 162)	LTS group (*n* = 39)	*p*-value
Gender, *n* (%)			0.006
Male	94 (58.0)	13 (33.3)	
Female	68 (42.0)	26 (66.7)	
Age (years), median (range)	56 (24–79)	56 (34–73)	0.750
Intravenous chemotherapy, *n* (%)			0.038
No	78 (48.1)	26 (66.7)	
Yes	84 (51.9)	13 (33.3)	
Previous surgical score, *n* (%)			0.016
0–1	53 (32.7)	21 (53.8)	
2–3	107 (66.0)	18 (46.2)	
NA	2 (1.2)		
KPS, *n* (%)			0.040
≥ 80	112 (69.1)	34 (87.2)	
< 80	28 (17.3)	2 (5.1)	
NA	22 (13.6)	3 (7.7)	
Pathological diagnosis, *n* (%)			0.000
Low grade	56 (34.6)	25 (64.1)	
High grade	59 (36.4)	11 (28.2)	
High grade with signet ring cells	41 (25.3)	1 (2.6)	
NA	6 (3.7)	2 (5.1)	
Vascular invasion, *n* (%)			0.096
No	143 (88.3)	39 (100.0)	
Yes	15 (9.3)	0 (0.0)	
NA	4 (2.5)	0 (0.0)	
Lymphatic metastasis, *n* (%)			0.048
No	139 (90.5)	39 (100.0)	
Yes	19 (7.5)	0 (0.0)	
NA	4 (1.9)	0 (0.0)	

*KPS* Karnofsky Performance Status score, *NA* not applicable, *LTS* long-term survival, *STS* short-term survival

### CRS + HIPEC characteristics

The CRS + HIPEC characteristics were compared between the two groups. In the STS group and the LTS group, PCI < 20 was 18 (11.1%) *vs. *10 (25.6%) (*P* = 0.020), and CC 0–1 was 42 (25.9%) *vs.*19 (48.7%) (*P* = 0.006) respectively. There were no statistically significant differences in the other indicators between the two groups, including operation duration, number of organ resection, blood loss, plasma transfusion volume, red blood cell transfusion volume, ascites volume, and number of anastomoses, etc (*P* > 0.05) (Table 2).

**Table 2:** Major CRS + HIPEC characteristics of PMP patients between STS and LTS groups

Variable	STS group (*n* = 162)	LTS group (*n* = 39)	*p*-value
Operative duration (min), median (range)	620 (24–990)	600 (120–1080)	0.538
Resected organs, median (range)			0.252
< 3	79 (48.8)	23 (59.0)	
≥ 3	83 (51.2)	16 (41.0)	
Anastomosis, *n* (%)			0.624
0	56 (34.6)	17 (43.6)	
1	66 (40.7)	14 (35.9)	
≥ 2	37 (22.8)	8 (20.5)	
NA	3 (1.9)	0 (0.0)	
PCI, *n* (%)			0.020
< 20	18 (11.1)	10 (25.6)	
≥ 20	143 (88.3)	29 (74.4)	
NA	1 (0.6)	0 (0.0)	
CC, *n* (%)			0.006
0–1	42 (25.9)	19 (48.7)	
2–3	118 (72.8)	20 (51.3)	
NA	2 (1.2)	0 (0.0)	
Stripped peritoneum area, median (range)	5 (0–9)	4 (0–9)	0.446
Blood loss volume (mL), median (range)	600 (50–5000)	500 (200–3500)	0.278
Fluid transfusion volume (mL), median (range)	6810 (1000–102,500)	6200 (2000–12,900)	0.074
Urine volume (mL), median (range)	1600 (400–5000)	1500 (600–5000)	0.694
RBC transfusion volume (U), median (range)	4 (0–20)	3 (0–16)	0.563
Plasma transfusion volume (mL), median (range)	800 (0–2000)	600 (0–1600)	0.797
Ascites volume (mL), median (range)	1300 (0–20,000)	1200 (0–10,000)	0.766
HIPEC regiment			0.776
120-mg cisplatin + docetaxel 120 mg	134 (82.7)	33 (84.6)	
120-mg cisplatin + mitomycin 30 mg	28 (17.3)	6 (15.4)	

*PCI* peritoneal cancer index, *CC* completeness of cytoreduction, *NA* not applicable, *RBC* red blood cells, *LTS* long-term survival, *STS* short-term survival

### Multivariate analysis

According to univariate analysis, there were statistical differences in the following 8 factors between the two groups (*P* < 0.05): gender, chemotherapy history, previous surgical score, KPS, pathological diagnosis, lymphatic metastasis, PCI, and CC score. The above factors were incorporated into the binary logistic regression model, and the results showed (Table 3) that CC 0–1 and low grade of pathological diagnosis were independently correlated with LTS of PMP patients.

**Table 3:** Multivariate analysis of PMP patients between STS and LTS groups

Variable	Wald	OR	95%CI	*p*-value
CC				
0–1 *vs*. 2–3	7.549	3.216	1.398–7.401	0.006
Pathological diagnosis				
Low grade *vs*. high grade	4.691	2.660	1.098–6.452	0.030
Low grade *vs*. high grade with signet ring cells	8.248	20.521	2.610–161.327	0.004

*OR* odds ratio, *CI* confidence interval, *CC* completeness of cytoreduction, *LTS* long-term survival, *STS* short-term survival

### SAEs

Among the 39 PMP patients with LTS, 7 (17.9%) cases had postoperative SAEs, including 2 (5.1%) cases involving the respiratory system, 3 (7.7%) cases involving the circulatory system, 3 (7.7%) cases involving the digestive system, and 4 (10.3%) cases involving infection. No deaths occurred within 30 days of surgery.

Among the 162 PMP patients with STS, 52 (32.1%) cases had postoperative SAEs, including 21 (13.0%) cases involving the respiratory system, 16 (9.9%) cases involving the circulatory system, 14 (8.6%) cases involving the digestive system, 24 (14.8%) cases involving infection, 2 (1.2%) cases involving the hematological system, 6 (3.7%) cases involving the urinary system, 2 (1.2%) cases involving the nervous system, and 4 (2.5%) cases died within 30 days after operation.

There was no statistically significant difference in the incidence of SAEs between the two groups (Table 4).

**Table 4:** SAEs between STS and LTS groups

Variable	STS group (*n* = 162)	LTS group (*n* = 39)	*p*-value
SAEs within 30 days after surgery			0.081
No	110 (67.9)	32 (82.1)	
Yes	52 (32.1)	7 (17.9)	

*LTS* long-term survival, *STS* short-term survival, *SAEs* serious adverse events

### Survival outcome

Among the 39 PMP patients with LTS, the median follow-up time was 148.2 (95% *CI*: 127.9–168.5) months, 11 (28.5%) died, 28 (71.8%) survived, and the median natural history was 138.9 (120.3–416.7) months. Of the 162 PMP patients with STS, 162 (100.0%) died, and 0 (0.0%) survived, and the median natural history was 29.2 (2.4–59.9) months.

## Discussion

This study compared the clinicopathological and treatment features of PMP patients between the STS group and LTS group. The results revealed that two crucial factors independently associated with LTS of PMP patients: CC score and pathological diagnosis. Interestingly, according to the OR value of multivariate analysis, the pathological diagnosis of PMP patients may be even more strongly correlated with LTS than the CC score.

At present, many clinical studies have explored the factors of survival benefit after CRS + HIPEC in PMP patients. Zhao et al. [10] retrospectively analyzed 453 PMP patients, and the result showed that PMP patients treated with CRS + HIPEC had seven independent prognostic factors, which were gender, previous surgical score, pathological diagnosis, lymphatic metastasis, PCI, CC, and splenectomy. Ansari et al. [13] studied 1000 PMP patients and showed that males, elevated CEA, elevated CA19.9, and high-grade pathological diagnosis predicted worse overall survival. The retrospective multicenter study of Chua et al. [14] on 2298 PMP patients showed that old age, SAEs, CC2-3, preoperative chemotherapy, and high-grade pathological diagnosis were independently associated with worse survival.

In this study, PMP patients with natural history ≥ 10 years were included in the LTS group, and those with natural history < 5 years were included in the STS group. These two groups may represent the extremes survival in PMP patients, and comparative analysis of the two groups is a rational approach to find the absolute factors of survival benefit in PMP patients. We identified two factors independently related to the LTS of PMP patients: one is the surgery-related parameter CC score, and the other is the pathological grade of the tumor. These results actually emphasize the importance of integrated treatment with CRS + HIPEC as the core for PMP patients. Before CRS + HIPEC, PMP patients should perform adequate preoperative preparation, exercise lung function, and receive enteral and intra intestinal nutrition support according to the nutritional status of the patient [15, 16]. During the operation, patient and careful operation were needed to achieve the maximum reduction of tumor cells and reduce the risk for adverse events. After CRS + HIPEC, great care should be taken to closely monitoring the changes of the patient’s condition, and individualized treatments should be given to prevent SAEs.

As a scoring system for the degree of tumor reduction, on the one hand, CC is related to the invasion degree of the tumor itself, and on the other hand, it is closely related to surgical techniques [17]. Complete CRS (CC0–1) depends not only on the patient’s tumor burden and physical status but also on the surgical ability and technique of the surgeon. Therefore, we need to consistently strengthen the surgical training of tumor surgeons in specialized peritoneal metastases treatment centers, so that more patients can achieve complete CRS. Polanco et al. [18] indicated that in their institution, approximately 180 and 90 CRS + HIPEC procedures are required to improve operative and oncologic outcomes for PM patients, respectively.

The pathological diagnosis of the tumor is related to the biological behavior and atypia degree of the tumor itself. There is a continuous need to explore the detailed pathological and physiological processes of PMP, to find some new molecular therapeutic targets or novel immunotherapy. For PMP patients diagnosed with high grade or high grade with signet ring cells, we need to explore a novel integrated treatment strategy to improve their relatively poor prognosis. The CRS + HIPEC is still the core but need combined with postoperative chemotherapy, molecular targeting treatment, or immunotherapy.

The study revealed that in STS and LTS groups, the proportion of patients with *PCI* ≥ 20 was 74.4% and 88.3%, respectively. Additionally, 46.2% and 66.0% of patients had a previous surgical score of 2–3. These findings indicate that some PMP patients even with high tumor burden can achieve LTS after receiving CRS + HIPEC treatment. However, this also highlights the existing diagnosis and treatment challenges of PMP in China; the majority of PMP patients do not receive professional integrated CRS + HIPEC treatment in the early stages of the disease, resulting in tumor continually progressing. Therefore, it is crucial to reduce the time from symptom onset or diagnosis to professional CRS + HIPEC treatment. Zhang et al. [19] analyzed the diagnosis and treatment status of PMP patients in China; their result showed that the median mistreatment time for PMP patients in China is approximately 15.3 months. Moreover, Yang et al. [6] emphasized that there need approximately 79 professional peritoneal metastases centers to meet the clinical needs of PMP patients in China. Significant progress remains to be made in achieving widespread implementation of PMP diagnosis and treatment, as well as the establishment of dedicated peritoneal metastases centers in China.

The disadvantages of this study are as follows: First, due to insufficient follow-up time, there are only 39 patients with natural history ≥ 10 years in our database, resulting in a small sample size in the LTS group; second, because lack of information related to postoperative treatment of patients, the detailed postoperative treatment strategy and characteristics of patients in the LTS group cannot be introduced; and third, this was a single-center retrospective case–control study with a moderate sample size, and higher-level studies must verify the conclusions.

## Conclusions

CC and pathological diagnosis were identified as crucial independent factors related to LTS of PMP patients. For PMP patients, the integrated treatment strategy with CRS + HIPEC as the core should be prioritized. It is essential to continuously enhance surgical training among oncologic surgeons. Furthermore, there is a need to further explore the pathophysiological mechanisms of PMP and develop more robust integrated treatment strategies.

## Data Availability

The datasets used and/or analyzed during the current study are available from the corresponding author on reasonable request.

## Abbreviations

PMP: Pseudomyxoma peritonei
CRS: Cytoreductive surgery
HIPEC: Hyperthermic intraperitoneal chemotherapy
LTS: Long-term survival
STS: Short-term survival
CC: Completeness of cytoreduction
PCI: Peritoneal cancer index
SAEs: Serious adverse events
KPS: Karnofsky Performance Status score
NA: Not applicable
RBC: Red blood cells
OR: Odds ratio
CI: Confidence interval
